# Supplementary material for: Systematic Review of Nutrition Interventions to Improve Short Term Outcomes in Head and Neck Cancer Patients
Source: Cancers (Basel). 2023 Jan 29;15(3):822. doi: 10.3390/cancers15030822 (PMC9913111; doi:10.3390/cancers15030822)
Supplement: Supplementary file 1 [file cancers-15-00822-s001.zip › cancers-2116666-supplementary.pdf]

Systematic Review of Nutrition Interventions to Improve Short Term Outcomes in Head and Neck Cancer Patients

Claire Leis, Anna E. Arthur, Xin Chen, Michael W. Greene and Andrew D. Frugé

Table S1. Additional characteristics of studies reporting nutrition interventions in patients with head and neck cancers.

	Number, Sex	Mean Age	Tumor Location	Stage	Anti-cancer Treatment
Sandmael et al., 2017	n = 41 (25 males, 16 females)	63.2 years	Nasopharynx, oropharynx, or hypo pharynx, larynx, and oral cavity		RT only: n = 24; CRT: n = 17
Cereda et al., 2018	n = 159 (114 males, 45 females)	63.8 year (Control)<br>66.5 year (Intervention)	Nasopharynx, oral cavity, oropharynx, parotid, hypopharynx, supralottis, larynx, laterocervical, thyroid	Stage 0: n = 5<br>Stage 1: n = 27<br>Stage 2: n = 44<br>Stage 3: n = 37<br>Stage 4: n = 46	RT: n = 159 (RT was administered to all patients)<br>Concurrent systemic therapy: n = 61
Jiang et al., 2018	n = 100 (69 males, 31 females)	48.18 years	Nasopharyngeal carcinoma	Stage 3: n = 43<br>Stage 4a-b: n = 57	Induction chemotherapy followed by CRT: n = 100
Britton et al., 2019	n = 307 (244 males, 63 females)	58 years	Nasopharynx, oropharynx, oral cavity, larynx, or hypopharynx	Stage 1: n = 12<br>Stage 2: n = 39<br>Stage 3: n = 57<br>Stage 4: n = 199	Concurrent chemotherapy: n = 147<br>Platinum-based: n = 205<br>Cetuximab: n = 40<br>Postoperative RT: n = 97
Orell et al., 2019	n = 58 (46 males, 12 females)	61 years	Oral cavity, oropharynx, hypopharynx, nasopharynx, or larynx	Stage 1: n = 2<br>Stage 2: n = 6<br>Stage 3: n = 12<br>Stage 4: n = 27	Surgery + Chemoradiotherapy: n = 11<br>Surgery + Radiotherapy: n = 2<br>Chemoradiotherapy: n = 41<br>Radiotherapy: n = 4
Lønbro et al., 2013	n = 30 (23 males, 7 females)	Median PROCR group: 56 years; Median PLA group: 59 years	Larynx (except glottic stage I + II), pharynx, oral cavity or in lymph nodes from an unknown primary tumor	Stage 1-2: n = 1<br>Stage 3-4: n = 20	RT alone: n = 10<br>CRT: n = 20
Capozzi et al., 2016	n = 60 (49 males, 11 females)	56.1 ± 9.2	Oral cavity, pharynx, larynx, paranasal sinuses, and salivary glands	Stage 1: n = 1<br>Stage 2: n = 7<br>Stage 3: n = 3<br>Stage 4: n = 48	RT alone: n = 15<br>CRT: n = 44

Table S2. Study Quality Assessment.

Criterion:	Sandmael	Cereda	Jiang	Britton	Orell	Lonbro	Capozzi	Mean	Standard Deviation
**1. Was there research question/hypothesis clearly stated?**	2, 2	2, 2	2, 2	2, 2	2, 2	2, 2	2, 2	2.0	0.00
**2. Were the inclusion and exclusion criteria clearly stated?**	2, 1 (2)	1, 1	2, 2	2, 2	2, 2	1, 1	1, 1	1.6	0.53
**3. Was a clear aim for the study stated?**	2, 2	2, 2	2, 2	2, 2	2, 2	2, 2	2, 2	2.0	0.00
**4. Were the methods of the study clearly described?**	2, 2	2, 2	2, 2	2, 2	2, 2	2, 2	2, 2	2.0	0.00
**5. Were the main findings (results) of the study clearly described?**	2, 2	2, 2	2, 2	1 ,2 (2)	2, 2	2, 2	2, 2	2.0	0.00
**6. Were study limitations discussed?**	2, 2	2, 2	2, 2	2, 2	1, 1	2, 2	2, 2	1.9	0.38
**7. Were outcomes (nutrition status, QOL, or treatment tolerance) identified via a validated measure?**	2, 2	2, 2	2, 2	2, 2	2, 2	2, 2	2, 2	2.0	0.00
**8. Was a sample size justification via power analysis provided?**	1, 0 (1)	2, 0 (2)	0, 0	2, 0 (2)	1, 2 (2)	2, 2	1, 0 (0)	1.3	0.95
**9. Are data analyses discussed?**	2, 2	2, 2	2, 2	2, 2	2, 2	2, 2	2, 2	2.0	0.00
**Total Score:**	17	17	16	18	17	17	15		

Scores for each criterion range from 0 to 2, depending on whether the criterion was unmentioned or unmet (0), partially met (1), or completely met (2). Reviewer 1 and 2 scores are listed with Reviewer 3 scores in parentheses for resolving discrepancies. The possible total study score ranges between 0 and 18. Higher total scores represent better quality.

Table S3. Supplements used in studies.

	Product Name:	Produced By:	Calories and Protein Provided:
Sandmael:	E+	Tine SA, Oslo, Norway	350 kcal, 7.5 g protein
Cereda	Resource Support Plus	Nestlé Health Science	500 kcal, 23 g protein
Jiang	Niufutai	EnterNutr, Guangdong, China	402 kcal, 18 g protein
Lonbro	In2Zym WPC80	LinusPro, Risskov, Denmark	120 kcal, 30 g protein

PubMed Search terms:

Search: **(((Nutrition Intervention) OR (Nutrition Counseling)) AND (Cancer)) AND (Head and neck cancer) AND (randomizedcontrolledtrial[Filter])**

("diet therapy"[MeSH Terms] OR ("diet"[All Fields] AND "therapy"[All Fields]) OR "diet therapy"[All Fields] OR ("nutrition"[All Fields] AND "intervention"[All Fields]) OR "nutrition intervention"[All Fields] OR (("nutrition s"[All Fields] OR "nutritional status"[MeSH Terms] OR ("nutritional"[All Fields] AND "status"[All Fields]) OR "nutritional status"[All Fields] OR "nutrition"[All Fields] OR "nutritional sciences"[MeSH Terms] OR ("nutritional"[All Fields] AND "sciences"[All Fields]) OR "nutritional sciences"[All Fields] OR "nutritional"[All Fields] OR "nutritionals"[All Fields] OR "nutritions"[All Fields] OR "nutritive"[All Fields]) AND ("counsel"[All Fields] OR "counseled"[All Fields] OR "counselings"[All Fields] OR "counselled"[All Fields] OR "counselling"[All Fields] OR "counseling"[MeSH Terms] OR "counseling"[All Fields] OR "counsellings"[All Fields] OR "counsels"[All Fields]))) AND ("cancer s"[All Fields] OR "cancerated"[All Fields] OR "canceration"[All Fields] OR "cancerization"[All Fields] OR "cancerized"[All Fields] OR "cancerous"[All Fields] OR "neoplasms"[MeSH Terms] OR "neoplasms"[All Fields] OR "cancer"[All Fields] OR "cancers"[All Fields]) AND ("head and neck neoplasms"[MeSH Terms] OR ("head"[All Fields] AND "neck"[All Fields] AND "neoplasms"[All Fields]) OR "head and neck neoplasms"[All Fields] OR ("head"[All Fields] AND "neck"[All Fields] AND "cancer"[All Fields]) OR "head and neck cancer"[All Fields]) AND "randomized controlled trial"[Publication Type]

Web of Science Search terms:
((((((TS = (nutritional counseling)) AND TS = (head and neck cancer)) OR TS = (Head and Neck Squamous Cell Cancer) AND SU = (Nutrition & Dietetics)) AND LA = (English)) AND DT = (Article)) AND (DT = = ("ARTICLE")) AND TS = (Body Composition))

Embase Search terms:
'nutrition intervention' AND ('head and neck cancer'/exp OR 'head and neck cancer')
